# Perception and Attitudes of Physicians and Nurses about Violence against Women

**DOI:** 10.1155/2013/785025

**Published:** 2013-03-21

**Authors:** Ana Cyntia Paulin Baraldi, Ana Maria de Almeida, Gleici Perdoná, Elisabeth Meloni Vieira, Manoel Antonio dos Santos

**Affiliations:** ^1^Departamento de Enfermagem, Faculdade de Ciências da Saúde, Universidade de Brasília, Brazil; ^2^Escola de Enfermagem de Ribeirão Preto, Universidade de São Paulo, Ribeirão Preto, SP, Brazil; ^3^Departamento de Medicina Social, Faculdade de Medicina de Ribeirão Preto, Universidade de São Paulo, Ribeirão Preto, SP, Brazil; ^4^Faculdade de Filosofia, Ciências e Letras de Ribeirão Preto, Universidade de São Paulo, Ribeirão Preto, SP, Brazil

## Abstract

Cross-sectional study compares the perception and attitudes about violence against women of physicians and nurses working in the primary health care clinics in Ribeirão Preto, SP. A total of 170 physicians and 51 nurses were interviewed in the District Health Clinics. Physicians feel more comfortable than nurses to talk about the sex life of patients (*P* = 0.048) and to investigate the use of drugs (0.001). Compared to the nurses greater number of physicians believed that the aggression to the woman by the husband should be treated as a medical problem (*P* = 0.000). Both believe that external factors, as alcohol or drug abuse, unemployment, and psychological problems of the husband and not of the victim, can cause violent acts. Most interviewees understand that gender violence exceeds the issues of individuality and privacy and has become a public health problem, by the dimension present in the social relationships.

## 1. Introduction

From the 1970s, violence against women (VAW) has become increasingly visible in Brazilian society. Since 1990, VAW is considered a topic of study and intervention in health assistance [1], and, as a reflection of national and international conventions, it is now considered a fundamental human right [2–7]. 

 Data from the World Bank show that domestic violence affects about 25% to 50% of women in Latin America, with costs of approximately 14.2% of Gross Domestic Product (GDP), which means US $ 168 billion [8]. In Brazil, it is estimated that every 15 seconds a woman is raped, usually in their home by a person who keeps loving relationship, and 70% of crimes against women happen at home and domestic violence costs to the country about 10, 5% of its GDP [8].

 Although the national political scene is favorable to combat VAW [9–11], its resolution involves the training of professionals who deal daily with these women.

 Even today, the use of health services by women in situations of violence, although frequent, is not very resolute, and most time it is ignored. For professionals, those cases are causes for frustration and sense of impotence [12], and this demand generates high costs with little result for the health system.

 Several factors have been identified as barriers to the recognition of VAW by health professionals, such as lack of training to identify women subjected to violence, ignorance about the handling of cases, feelings of insecurity when dealing with the problem, and little support for the victims [13, 14]. Adequate assistance is hindered by the spread of the erroneous idea that intimate partner violence is a private matter that should be resolved by involved ones [15] and also by the common sense that domestic violence is a problem of public security and justice spheres [16].

 A qualitative study conducted in Ribeirão Preto, SP, Brazil, showed that physicians are able to identify a woman in a violent situation through physical marks and other somatic symptoms associated with the event, and that they know some procedures for referral of those cases. However, they often ignore the significance of giving them the expected routing; among the reasons for not referring those cases, discrediting the woman will take action; feeling of helplessness and fear of the abuser to react against professional are mentioned [17, 18].

 Assistants' perception of violent situations is constructed in the course of their professional and personal life, depending on intrinsic and extrinsic factors of their personalities. This perception interferes directly in the care provided to victims. Nurses and physicians are expected to present with an empathic attitude, free of preconceptions and stigmas, furnishing adequate care for women living in violent situations. Those professionals play a major role in caring for victims of violence, as the first to come into contact with those women in health services and generally maintain a long-term relationship with users. However, the literature has demonstrated the lack of education and training for professionals on the subject [19–26].

 Any proposal for intervention in order to sensitize those professionals for the proper handling of cases of gender violence should be planned according to reality they need to transform. Therefore, it is necessary to know the characteristics of the professionals involved in caring for victims of domestic violence, their personal concepts, and perceptions about the issue.

 Therefore, this study aimed to conduct a comparative analysis of perceptions of physicians and nurses on the phenomenon of VAW in primary health care system of Ribeirão Preto, SP, Brazil. From this analysis, we come to know and meet the specific needs of each professional training, to better accommodate the cases of VAW and direct actions of prevention and combat.

## 2. Methods

 This is a quantitative, cross-sectional, and comparative survey, including all physicians (*n* = 170) and nurses (*n* = 51) from district basic health units (UBDS) in Ribeirão Preto, SP, Brazil, who were questioned about their perceptions and attitudes towards VAW.

 Data collection instrument used was a modified version of a questionnaire used in a study conducted with residents and medical students, also from Ribeirão Preto, SP, Brazil [17], complemented by issues raised in the literature review [27]. After a pretest necessary adaptations in questionnaire were carried out, and the final instrument contained 17 questions related to feelings, attitudes, and personal viewpoints of professionals in relation to the subject being studied and applied face to face by trained interviewers for this purpose. The issues are outlined in the tables presented in the results.

 The study was approved by the Municipal Health Department of Ribeirão Preto, SP, Brazil, and later by the Ethics Committee in Research of the University Hospital, Faculty of Medicine, University of São Paulo (USP-HCFMRP). Professionals were all provided guidance on the study, including the confidentiality of information provided and voluntary aspect of participation. Those who agreed to participate signed a consent form, according to the norms of Resolution 196/96 of the National Health Council, Ministry of Health, Brazil.

 Data were analyzed using the statistical package Stata 9.0 (StataCorp LP, TX, USA); association between variables was assessed by the chi-square test and Fisher's exact test; the association hypothesis was accepted with *P* less than or equal to 0.05.

 Study limitations: the data show the reality of professionals that works in the UBDS of Ribeirão Preto, not being possible its application to other populations.

## 3. Results

 We interviewed 221 health professionals, 51 nurses (23.08%) and 170 physicians (76.92%). Among the nurses, five were male (9.8%), and 46 were female (90.2%), whereas among physicians, 114 were men (%67.05), and 56 women (32.95%) (*P* < 0.001). The age ranged from 24 to 65 years with a mean age of 38.6 years and median of 36.5 years, respectively. Most respondents declared themselves as white and married, and nurses had more years of service (mean 18.04 years) than physicians (mean 10.9 years).

 Most professionals showed to be comfortable about talking with the patient on diverse subjects. However, 20 nurses (39.22%) and 54 physicians (31.76%) reported to feel uncomfortable to ask patients about domestic violence. It was observed that seven nurses (13.73%) and 17 physicians (10%) reported they had never asked their patients about the existence of violent relationships at home (Table 1).

Physicians showed to feel more comfortable than nurses to discuss issues such as patient's sexuality (*P* = 0.048) and use of illicit drugs (*P* = 0.001).

 Regarding perception of professionals about gender violence, respondents were asked whether they believed that women who live in situations of violence may have some secondary gain to justify remaining under aggression. Although majority of nurses (80%) and physicians (65.88%) have disagreed with that statement, and some professionals considered that as a real situation (*P* = 0.058).

 Professionals interviewed believed that the role of the physician in case of women in situations of violence must be similar to that played for children victimized—38 nurses (74.51%) and 116 physicians (68.23%) agreed with this statement. Nevertheless, opinions on the attribution of this role differed: majority of nurses (54.9%) disagreed that the assault on woman by her husband should be treated as a medical problem; physicians, however, agreed with this statement (72.94%) (*P* < 0.001) (Table 2).

Most nurses and physicians believed that violence can be caused by external factors such as unemployment (66.7% and 69.4%, resp.) and abuse of alcohol and drugs (94.1% and 91.7%, resp.). Both groups agreed (66.7% of nurses and 75.3% of physicians) that the psychological problems of the husband may be responsible for aggression.

 Respondents were almost unanimous in denying the statement that raped women remain in violent environment because of masochism (90.2% of nurses and 93.5% of physicians) or in accepting husband's violence as a reaction to provocation (96.0% and 97.6% resp.). 46 nurses (90.2%) and 136 physicians (80%) did not consider aggression by husband as a matter of intimacy or privacy. Finally, considering punishment of the offender, 39 nurses (76.5%) and 127 physicians (74.7%) agreed that they should be arrested for assault.

## 4. Discussion

 The World Report on Violence and Health detaches that comfort of the interviewer to talk about the issue of violence with his patient ensures greater success in the identification of aggression [4]. In general, most practitioners reported feeling comfortable about talking to the users of health services about delicate issues, including domestic violence. However, this was the topic on which most professionals felt uncomfortable. In a similar study conducted in Seattle, United States, fewer physicians (23.5%) and more nurses (50.7%) reported discomfort in addressing the issue [28] and in the United Kingdom, the percentage of professionals who said they feel uncomfortable was 44% [29].

 Schraiber and D'Oliveira [29] pointed out that a situation that affects 20–50% of women cannot be subject to stigma or shame, and fear of professionals to address the subject often expresses a self-moral judgment, and not a constraint in exposing situation by users. The professional must be able to provide adequate care to the patient, regardless of their personal beliefs or preconceived ideas. Some authors [30, 31] have emphasized that the low solving and a sense of powerlessness in the face of violence lead us to guard against contact with such adverse situations.

 Domestic violence can also bring out individual emotions and unresolved conflicts, and health professionals are not exempt from suffering such violence. Thus, some studies [30, 32–34] have identified that the prevalence of VAW among nurses is higher than the prevalence among women in the general population and higher than among physicians. Moreover, the converse situation also can be a barrier which helps to promote professionals discomfort. Thus, those who have never experienced such a situation may present difficulties in coping.

 This fact can cause two distinct consequences in the care of women victims of domestic violence: an empathic attitude by the nurse, with involvement with the case and commitment to forwarding it, or an attitude of detachment, for fear of bringing up memories and feelings that could cause deep suffering.

 Physicians were more comfortable than nurses to talk about the sexual life of patients and use of illicit drugs, as previously reported [28]. The ease in addressing the above topics could be justified by the way the division of attributions in health services is organized: while nurses are in charge of or nursing care, administrative and bureaucratic functions, the role of physicians includes basically service to individual queries due to high demand of the population for this type of service. As information on alcohol and illicit drug use is essential to make a correct diagnosis of the health situation of the patient, it is expected that these professionals develop greater skill and mastery to address such issues.

 Interestingly, 13.7% of nurses and 10% of physicians never inquired the patients about possible situations of gender violence. Although these percentages are lower than the 23% to 32% found in literature [20, 28, 30, 35], it may suggest that, for some professionals, research and diagnosis of cases of violence by the intimate partner are absolutely unnecessary.

 Surveys in Canada and the United States found a higher percentage of nurses than of physicians who had never identified a case of gender violence [28, 30], but in our study the difference between the two professional groups was not significant.

Although not the majority, an important amount of nurses and physicians believed that women living in situations of violence have secondary gains and therefore remain in this situation, demonstrating the cultural devaluation of women and gender prejudices that exist in our society.

 When professionals faced with cases where there was suspicion of gender violence, the majority tended to infantilize the victim, which demonstrates its agreement with the statement that his role in the case of victimized women must be equal to that played for victimized children. Data are consistent with a similar study conducted in Africa, where 78.1% of physicians stated that the two situations should be similarly handled [36].

 According to some authors [30], women are treated as dependent subjects and infantilized as social subjects. Thus, developing equal roles for the care of women and children is to consider that the woman is still a subject in education that needs a “tutor” to take decisions and actions that she alone would be unable to perform.

 Physicians believed that the assault on woman by her husband should be treated as a medical problem, while the nurses did not agree with this statement. As previously stated [30], health professionals tend to recognize domestic violence as part of the scope of justice and public safety; health services would be responsible only for treating injuries.

 The fact that gender violence is part of the curricular content of the graduation course of most physicians interviewed could explain the clarity of their professional role opposing VAW. Still, the percentage of physicians who agreed with the statement mentioned above in this study (72.94%) is lower than that found in the literature—around 90% [20, 27, 36]. Nurses interviewed by Aksan and Aksu [20] also agreed that intimate partner violence should be treated as a medical problem, unlike our results. Search with physicians and residents in Ribeirão Preto, SP, Brazil [27], found that physicians belief in their investigative role on gender violence was directly associated with greater knowledge on the topic.

 Not considering domestic violence as a medical issue hurts ethical principles of beneficence and nonmaleficence. Beneficence because, once diagnosing and acting to help the victim to free herself from violence, it avoids continuation and aggravation of the case; it benefits the patient. The principle of nonmaleficence is also applicable because the failure to diagnose it causes damage to victims [15].

Beliefs about the factors that may be causes of violence did not differ between the two professional groups: physicians and nurses believed that external factors such as abuse of alcohol/drugs, unemployment, and psychological problems of husband and not the victim can trigger violent acts. Our results are similar to those found among physicians in South Africa [36]. Among nurses, the percentage of professionals in our study (94.12%) who believed that violence is caused by alcohol and other drugs by intimate partners is greater than that found among nurses in Finland 62% [37].

 It is known that factors such as alcohol/drugs, unemployment, and psychological problems often provide violent acts [38]. However, it may not be considered to be the only cause of the VAW, because sober men also attack, and often alcohol is used as a cover for violent behavior [14].

 The statements that emphasized the dominance of men in society have been denied by almost 90% of respondents in both groups of professionals. For 92% of American nurses in the study of Bessette and Peterson [23] there was no justification for violence against women, a percentage similar to that found in this study. Among physicians, however, we realized that gender preconception is higher than that found among physicians in South Africa [36].

 Those stereotypes influence the way society reacts to denouncements [36] and may even increase the difficulty of raped women to report the abuse, given the fear of being despised by those around them.

 Violence against women meets “justification” in social norms based on gender relations, that is, rules that reinforce an appreciation for different male and female roles. What changes from country to country are the reasons given for adopting this type of violence.

Although the respondents in our study have shown a positive stance in relation to gender differences, more studies are needed, allowing greater depth of analysis of the discourses of professionals. In a qualitative study with gynecologists who serve at basic districts in Ribeirão Preto, SP, Brazil, it was found that, although they recognize female social achievements, those professionals were still trapped in a sexist model by assigning responsibility for the maintenance of this model to the women themselves [38].

 The percentage of physicians in this study responded that violence has intimate, and private character was greater than that found among South African physicians, but lower than those obtained in Turkey [20] and México [35]. Understanding VAW as an event of privacy and intimacy means not recognizing it as part of their field and therefore does not act to prevent it, identify it, and fight it.

## 5. Conclusions

 We can conclude that, in general, physicians and nurses interviewed in our study affirmed to feel comfortable to talk about gender violence and have a positive perception of gender differences, but they tend to infantilize victimized women.

This study could be used as a diagnostic tool to think about possibilities of intervention for this population.

## Figures and Tables

**Table 1:** Distribution of physicians and nurses from district basic health units of Ribeirão Preto, SP, according to their feelings to talk to the patient on any subjects.

Health professionals' feelings to talk to the patient according to different subjects	Uncomfortable	%	Comfortable	%	Never asked	%	*P *	Total (%)
(a) About alcohol consumption								
Nurses	4	7,84	47	92,16	—	—		100
Physicians	8	4,71	162	95,29	—	—		100
(b) About smoking								
Nurses	—	—	50	98,04	1	1,96		100
Physicians	2	1,18	168	98,82	—	—		100
(c) About sexual life								
Nurses	14	27,45	32	62,75	05	9,80	0,048	100
Physicians	29	17,06	139	81,76	02	1,18		100
(d) About illegal drugs consumption								
Nurses	20	39,22	30	58,82	1	1,96	0,001	100
Physicians	30	17,65	139	81,76	1	0,59		100
(e) About violent relationship with her partner								
Nurses	20	39,22	24	47,05	7	13,73		100
Physicians	54	31,76	99	58,24	17	10		100

Database: district basic health units of Ribeirão Preto, SP, Brazil, 2008 [18].

**Table 2:** Distribution of physicians and nurses from district basic health units of Ribeirão Preto, SP, according to their perception in cases of suspected intimate partner violence.

Professional's perception in case of suspected intimate partners violence.	Agree	%	Indifferent	%	Disagree	%	*P *	Total (%)
(a) The role of physicians in case of victimized women should be the same role that they take in case of victimized child.								
Nurses	38	74,51	—	0	13	25,49		100
Physicians	116	68,23	04	2,35	50	29,42		100
(b) The violence by women's husband should be seen and treated as a doctor problem.								
Nurses	23	45,1	—	0	28	54,9	**0,00**	100
Physicians	124	72,94	06	3,53	40	23,53		100
(c) The violence by women's husband is caused by social problems such as unemployment.								
Nurses	34	66,67	03	5,88	14	27,45		100
Physicians	118	69,41	11	6,47	41	24,12		100
(d) The violence by women's husband is caused by drug and alcohol abuse.								
Nurses	48	94,12	01	1,96	02	3,92		100
Physicians	156	91,76	05	2,95	09	5,29		100
(e) The violence by women's husband is caused by psychological problems of the victim.								
Nurses	11	21,57	05	9,8	35	68,63		100
Physicians	38	22,36	09	5,29	123	72,35		100
(f) The violence by women's husband is caused by psychological problems of the husband.								
Nurses	34	66,67	07	13,72	10	19,61		100
Physicians	128	75,29	16	9,42	26	15,29		100
(g) The women attacked by their husband or partners remain in this situation because of their masochism.								
Nurses	04	7,84	01	1,96	46	90,2		100
Physicians	07	4,11	04	2,36	159	93,53		100
h) The violent husbands should have compassion, because they feel emotional upset.								
Nurses	05	9,8	02	3,92	44	86,28		100
Physicians	10	5,88	10	5,88	150	88,24		100

Database: district basic health units of Ribeirão Preto, SP, Brazil, 2008 [18].
